# Social Gradients and Physical Activity Trends in an Obesogenic Dietary Pattern: Cross-Sectional Analysis of the UK National Diet and Nutrition Survey 2008–2014

**DOI:** 10.3390/nu10040388

**Published:** 2018-03-22

**Authors:** Laura Johnson, Zoi Toumpakari, Angeliki Papadaki

**Affiliations:** Centre for Exercise, Nutrition and Health Sciences, School for Policy Studies, University of Bristol, 8 Priory Road, Bristol BS8 1TZ, UK; z.toumpakari@bristol.ac.uk (Z.T.); angeliki.papadaki@bristol.ac.uk (A.P.)

**Keywords:** dietary patterns, obesogenic, reduced rank regression, National Diet and Nutrition Survey

## Abstract

An energy-dense, high-fat, low-fibre dietary pattern has been prospectively associated with the development of obesity in childhood but is population-specific, which limits translating the pattern into interventions. We explored the generalisability and correlates of this obesogenic dietary pattern in the UK National Diet and Nutrition Survey (NDNS) for the first time. Data came from participants (*n* = 4636 children and *n* = 4738 adults) with 4-day food diaries in NDNS 2008–2014. Reduced rank regression was applied to 51 food groups to explain variation in energy density, fibre and fat intake. Consistency of the pattern in population subgroups (according to sex, age, occupation and income) was compared with the whole sample pattern using coefficients of congruence (COC). Pattern correlates (sociodemographic, survey year, physical activity and eating related behaviours) were explored using multiple linear regression. Food group loadings were similar to the previously identified obesogenic dietary pattern and were generalisable across all sub-groups (COC: 0.93–0.99). An obesogenic diet was associated with eating takeaways, being omnivorous, a manual household occupation and lower household income in both adults and children (*p* < 0.0001). Dieting for weight loss, being older, more physically active and less sedentary was associated with a less obesogenic diet among adults (*p* < 0.0001). Future experimental studies should investigate if changes in this obesogenic pattern could be used to monitor the effectiveness of obesity prevention policies or develop personalised interventions.

## 1. Introduction

The prevalence of adult and childhood obesity has risen dramatically representing an important global public health problem associated with chronic disease, mortality and disability [1]. In the United Kingdom (UK), more than a quarter of adults and a fifth of school children are obese, [2]. In England alone, obesity results in more than 30,000 deaths and costs the National Health Service approximately £6.1 billion every year [3]. Diet is a key modifiable risk factor for obesity [2]. A wide range of dietary factors have been implicated, including dietary fat, fibre and energy density [4]. People, however, do not consume nutrients or foods in isolation. Nutrients are combined within foods and foods are eaten in combinations within a whole dietary pattern. The importance of studying dietary patterns has been well established as providing more useful insights into associations of diet with health outcomes, including obesity, compared to individual dietary components [5]. Dietary patterns can be defined as a priori (e.g., indices measuring adherence to established diet-disease hypotheses) or a posteriori methods (e.g., data-driven analyses, which identify common combinations of foods or groups of individuals with similar diets that may or may not relate to health) [5]. Reduced rank regression (RRR) is a hybrid approach for identifying novel disease-specific dietary patterns that combines data-driven analysis with prior knowledge of disease mechanisms. RRR refines common dietary patterns (maximised to explain all food intake variation) to further explain variation in specified intermediate variables e.g., nutrients, hypothesised to be on the causal pathway from diet to disease. Thus, a pattern that is both consumed within a population and relevant to the disease of interest is identified [6].

RRR has previously been applied to identify obesogenic dietary patterns, in both adults and children. For example, a dietary pattern characterised by low-fat, high-carbohydrate, and high-fibre foods was associated with lower body weight gain in adults in the German EPIC-Potsdam cohort study [7]. Also, a higher energy-dense, high-fat and low-fibre dietary pattern score was associated with an increase in waist circumference in Australian girls (from 14 to 17 years) [8]. In the UK, a dietary pattern explaining higher carbohydrate/fibre and lower fat foods was associated with lower body mass index (BMI) in 1238 participants of the 1946 Birth Cohort followed from age 36 to 43 to 53 years [9]. Previously, we have shown that an energy-dense, high-fat, low-fibre dietary pattern, characterised by high intakes of low-fibre bread, crisps, sweets, chocolate, biscuits and processed meats in tandem with low intakes of fruits, vegetables, high-fibre bread and breakfast cereals, was associated with the development of excess adiposity in children from age 5 and 7, to 9 years in the Avon Longitudinal Study of Parents and Children (ALSPAC) [10]. The same pattern tracks with age and is also associated with further fat mass gains from age 11–15 years, indicating that this specific combination of foods intakes in childhood is associated with adiposity in adolescence [11]. Findings from these longitudinal studies support the potential for RRR-derived dietary patterns, and particularly an energy-dense, low-fibre, high-fat pattern, as a basis for personalised interventions or monitoring the effect of policies to prevent obesity.

A common problem of data-driven methods is that the diet patterns are sample-specific (i.e., the relative importance of foods rich in energy density and fat and low in dietary fibre may be unique in each sample). Thus, the importance of specific foods characterising an obesogenic dietary pattern may vary over time or between locations, age or socioeconomic groups. Obesity prevention efforts could utilise the pattern loadings for food groups previously identified for an obesogenic dietary pattern e.g., in ALSPAC, but this combination of foods may only be relevant to children in South West England at the turn of the century and not the rest of the UK. Identifying food loadings for an obesogenic dietary pattern in a nationally representative sample of the UK population is an essential step to confirm if the pattern is stable. Therefore, we aimed to apply RRR in the UK National Diet and Nutrition Survey (NDNS) for the first time. We repeated the RRR in different subgroups to explore the generalisability of the pattern. Finally, we investigated the correlates of the pattern in order to inform the need for targeted (i.e., to specific population groups) and tailored (i.e., by tackling the consumption of specific foods) interventions to change the obesogenicity of diets in the UK.

## 2. Materials and Methods 

Data were from 4636 children (≤18 years) and 4738 adults (19–64 years) from waves 1–6 (February 2008–August 2014) of the UK NDNS. More details on the design of NDNS can be found elsewhere [12]. In brief, it is an annual programme of cross-sectional surveys assessing the dietary intake and nutritional status of the general UK population aged 1.5 years and over. The sample is drawn from a selection of postcodes across the UK, which are divided into Primary Sampling Units (PSUs). Households are randomly sampled within PSUs and 1 adult (19 years+) and 1 child (1.5 to 18 years) are invited to participate. In some households, only children were asked to take part to ensure an adequate sample of children are available for comparisons. Participants completed a consecutive 4-day food diary. Participants with at least 3 days were subsequently visited by a nurse who conducted anthropometric measurements and collected a blood sample. NDNS was conducted according to the Declaration of Helsinki guidelines and all procedures involving human subjects were approved by the Oxfordshire A Research Ethics Committee. All participants provided written informed consent. Data for the current analysis were downloaded from the UK data archive [13].

Food diaries were self-reported by participants aged 12 years and over and parent-reported for children under 12 years old. Portion sizes were estimated with household measures, portion size photographs and food weights from labels. For homemade dishes, participants recorded recipes on a separate page. Diaries were coded using DINO (Diet In Nutrients Out) [14]. For the current analysis, NDNS food items were harmonised into 51 pre-defined food groups previously used for dietary pattern analysis in ALSPAC [10]. New food groups that were not consumed in ALSPAC but were in NDNS were created including alcoholic beverages and infant/toddler foods and drinks (Supplementary Information 1). Each participants’ average intakes (g/day) for each food group were calculated from the food level file. Data on average total daily fat, fibre and energy intakes were used from the nutrients file and converted to % total energy from fat ((kcal from fat/total kcal) * 100) and fibre density (total fibre (g)/total energy intake (1000 kcal)). Dietary energy density was computed directly from the food level file as total kcal/total grams for foods only excluding drinks because they spuriously dilute estimates [15]. The extent of dietary misreporting was assessed using an individualised method [16] based on the ratio of energy intake (EI) to total energy expenditure (TEE). TEE was calculated from equations derived from doubly labelled water (DLW) studies in children and adults [17,18]. To account for random error in estimating EI and TEE, a 95% confidence interval of the ratio EI/TEE was calculated. Participants falling within the 95% CI (EI/TEE = 0.63 to 1.34 for participants with 3 days completed or 0.65 to 1.35 for participants with 4 days completed) were classified as plausible reporters, while those below or above the 95% CI were classified as under-reporters and over-reporters respectively. A categorical variable indicating under-, plausible-, and over-reporting was created and used in analyses. 

Measured weight (kg) and height (cm) were used to calculate BMI (kg/m^2^). For children 1.5–2 years, length was measured instead of height. BMI groups (normal weight, overweight and obese) were created using standard World Health Organisation cut-offs for adults (normal weight BMI 18–24.99 kg/m^2^, overweight BMI 25–29.99kg/m^2^ and obese BMI 30kg/m^2^ or more). For children, international (IOTF) criteria [19] (<18 years) were used to define weight status and BMI was also standardised for age and sex based on the 1990 British Growth Reference (UK90) [20]. 

Physical activity in adults (>16 years) was estimated with the validated Recent Physical Activity Questionnaire (RPAQ) [21], which assessed type, amount of PA and screen time from Year 2 of the rolling programme. Time spent in moderate or vigorous PA (MVPA) was summed to provide daily MVPA (min/day). Physical activity data in children was measured using waist-worn Actigraph accelerometers worn for 7 days concurrent to diary completion. Data were available in just 12% of the sample in years 1 and 2, therefore, these data were not used initially but have been incorporated for supplementary analyses in response to review. The time spent watching media on TV or tablets and time spent using a computer on weekdays and weekend was reported by adults in the RPAQ. Daily time spent on each of these sedentary activities (h/day) were calculated and were used in analyses. 

Eating-related behaviours including the frequency of eating meals out and having takeaways at home was obtained in interviews by asking ‘On average, how often do you/does child eat meals out in a restaurant or café?’ and ‘On average, how often do you/does child eat take-away meals at home?’, respectively. Response options ranged from ‘rarely/never’ to ‘≥5 times per week’ and were further collapsed for analysis (≥1 per week vs. <1 per week). Information was also obtained on whether participants followed a vegetarian diet (yes/no) and whether they were on a weight loss diet (yes/no). Socioeconomic position was represented by household occupational social class and income, which were self-reported during the interview. Each participant was assigned a socio-economic classification based on the occupation of the householder with the highest income.

## 3. Statistical Analyses

Dietary patterns were derived with Reduced Rank Regression (RRR). To replicate the obesogenic dietary pattern previously identified in ALSPAC, we used dietary energy density (DED), fibre density (FD) and total fat as a percentage of energy intake (% fat) as response variables [10,11,22]. A total of 51 food groups were used as predictors, to generate 3 dietary patterns (based on the number of response variables). RRR calculates individual dietary pattern scores, based on a linear combination of all food group intakes weighted by pattern loadings. RRR generates pattern loadings for each food group, which reflects the contribution of each food group to the overall dietary pattern. Positive pattern loadings increase the dietary pattern score and negative pattern loadings reduce the score. The first pattern explained 44% of response variation, while the remaining two explained less than 20% variation combined. Therefore, the first dietary pattern was retained for subsequent analyses. The generalisability of the dietary pattern derived in different populations, i.e., gender, age, occupation, household income, was assessed with the Tucker’s coefficient of congruence (COC) to understand whether a single overall dietary pattern score could adequately represent the entire UK population. For details on calculation of a COC see Supplementary Information 2. A COC from 0.85–0.94 suggests fair similarity, and over 0.94 suggests the patterns are essentially equal. A COC below 0.85 indicates that the overall dietary pattern is not adequately representative for that sub-group therefore specific patterns should be considered [23]. To provide a baseline for comparison RRR was run twice following a random split sample approach to derive a COC (φ = 1.00, Supplementary Table S1). 

Survey weights provided by NDNS were used in all descriptive and trend analyses to account for participant non-selection and non-response. The use of survey weights means that only percentages (95% confidence intervals (CIs)) are presented rather than raw frequencies. Variables were described with the use of mean and 95% CIs if normally distributed or with the use of median and interquartile range (IQR) otherwise. Correlation between two continuous variables was assessed with Pearson’s *r* correlation coefficient. Stratified analysis by age (children ≤18 years and adults >18 years) was used to examine trends in dietary pattern score after interaction tests confirmed different associations were likely (*p* < 0.05). Separate quintiles of dietary pattern score were generated for children and adults. The distribution of dietary variables across quintiles of dietary pattern score was assessed with linear regression models adjusted for age, gender and energy intake. Weighed percentages of people consuming each food group within each quintile of the dietary pattern score were generated to test trends in consumption of foods with the highest positive and negative pattern loadings because most food groups were not normally distributed (therefore a mean (95% CI) was not appropriate). 

Due to 13% of participants not providing information on household income, as part of sensitivity analysis, RRR was run for participants with missing vs. non-missing income information. Dietary patterns generated between the two groups were equal based on the COC (φ = 0.99), and there was little evidence of difference in the dietary pattern score between the two groups (*p* = 0.09). All analyses were performed in Stata Statistical Software: Release 13 (StataCorp LP., College Station, TX, USA); code used to perform the RRR in Stata is provided in Supplementary Information 3. 

## 4. Results

The sample comprised 4636 children aged 1–18 years, who were 51% male, 43% from families with a professional occupation, and 57% had a household income of less than £23,000 per year (Table 1). Most children were of healthy weight (76%), with a mean BMI standard deviation score (SDS) 0.5 (95% CI 0.4, 0.5) and were primarily classified as having plausible reports of energy intake (79%). There were 4738 adults aged 19–96 years, 44% had a professional occupation, and 46% earned a household income of less than £23,000 per year. Most adults were overweight or obese, with a mean BMI of 27.4 (95% CI 27.2, 27.6) kg/m^2^. Most adults were classified as plausible reporters (71%). Adults spent a median 3 h/day watching media on TV or tablets, 1h/day using computers and 36 min/day in moderate-to-vigorous physical activity.

**Figure 1:**
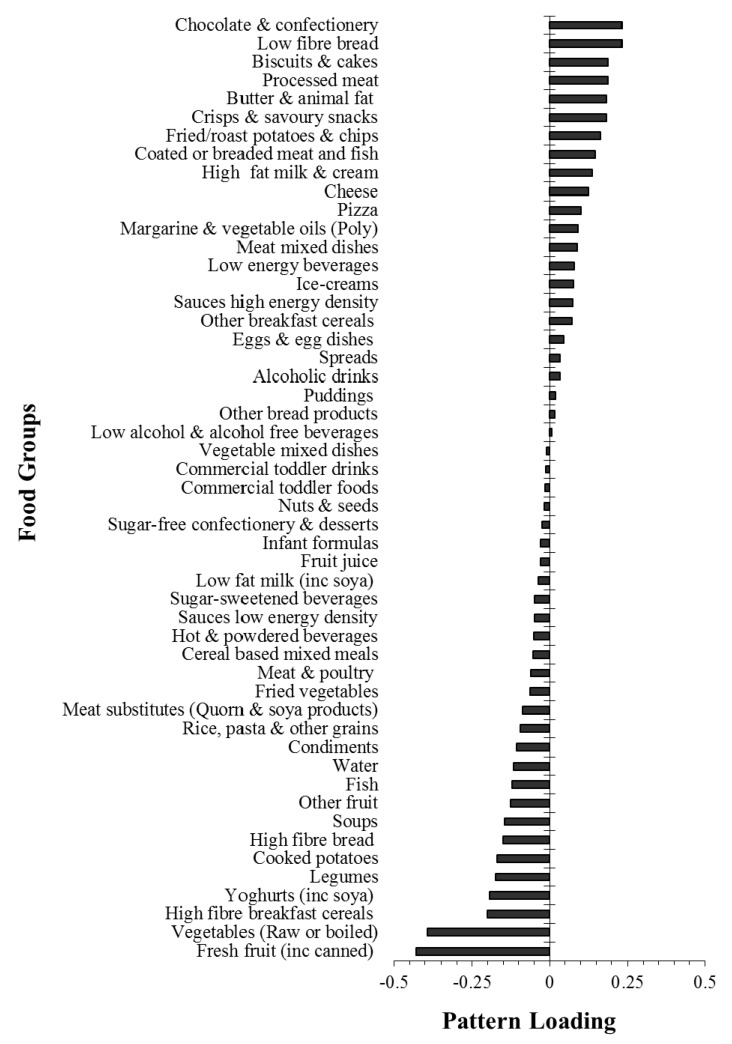
Dietary pattern loadings for each food group in children and adults.

The obesogenic dietary pattern derived in the whole sample explained a total of 44% of response variation, including individually 59% DED, 45% FD and 28% %fat variation. A high pattern score represents an energy-dense, low-fibre, high-fat diet with high correlations with DED (r = 0.77) and FD (r = −0.67) and a moderate correlation with %fat (r = 0.53). The pattern loadings for food groups are displayed in Figure 1. ‘Fresh fruit’ and ‘Vegetables (raw or boiled)’, had the largest negative pattern loadings followed by ‘High fibre breakfast cereals’, ‘Yoghurts’ and ‘Legumes’, indicating that low consumption of these food groups contributed most to a high obesogenic pattern score. In contrast, ‘Chocolate and confectionery’, ‘Low fibre bread’, ‘Biscuits and cakes’, ‘Processed meat’ and ‘Butter & animal fat’ had the highest positive loadings meaning increased intake of these foods contributed to a more obesogenic dietary pattern score. 

The generalisability of the dietary pattern was confirmed by the replication of food group loadings in a range of sub-groups. Coefficients of congruence (range 0.93 to 1.00), comparing pattern loadings in each sub-group with the overall sample, are displayed in Supplementary Table S1. The lowest congruence was observed for adolescents (0.93); where the amount of variation in fibre was lower for adolescents vs. the whole sample (19 vs. 45%). While still indicating overall good similarity, some age-related differences in pattern loadings for a small number of food groups were observed (Supplementary Figure S1). Sugar sweetened drinks and cheese had larger negative and slightly higher positive loadings for adolescents respectively. Low fibre bread contributed more to the pattern among adults vs. children; Butter had a higher loading among those aged 65+ years; High-fat milk and cream were more important among the 1–5 age group and low energy beverages contributed more to an obesogenic pattern score among children vs. adults (Supplementary Figure S1).

Supplementary Table S2 and Table S3 display variation in key nutrients and food groups by quintile of dietary pattern score for children and adults, respectively. For both children and adults, dietary energy density differed by approximately 3 kJ/g, fibre density differed by approximately 1g/MJ and % fat intake differed by 8–9% from quintile 1–5 of the obesogenic dietary pattern score. Self-reported total energy intake was around 220–300 kcal higher in quintile 5 vs. 1 among children and adults respectively. The percentage of consumers of foods with negative loadings decreased from quintile 1–5, for example 88% of children in quintile 1 consumed fruit compared with just 62% in quintile 5. More than 90% of adults consumed vegetables regardless of dietary pattern score quintile, suggesting the main differences in this food group were driven by the amount, rather than if vegetables were consumed. Mean (95% CI) vegetable intake was 75 (95% CI 70, 80) grams/day in quintile 5 vs. 201 (193, 209) grams/day in quintile 1 of the dietary pattern score, equating to a difference of approximately 1.5 servings a day.

**Figure 2:**
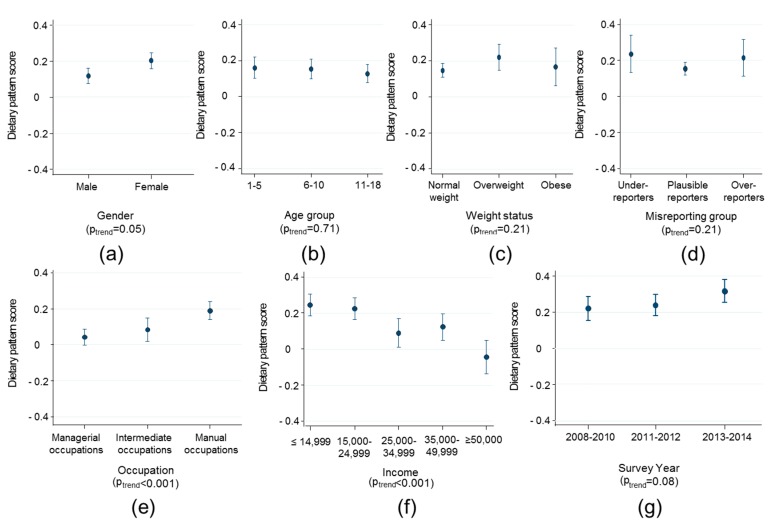
Trends in the obesogenic dietary pattern score among children (<18 years) by (**a**) sex; (**b**) age; (**c**) weight status; (**d**) energy reporting status; (**e**) occupation; (**f**) household income; and (**g**) survey year. Figures present marginal means and tests for trend with survey-weighted linear regression adjusted for age, gender and daily energy intake.

**Figure 3:**
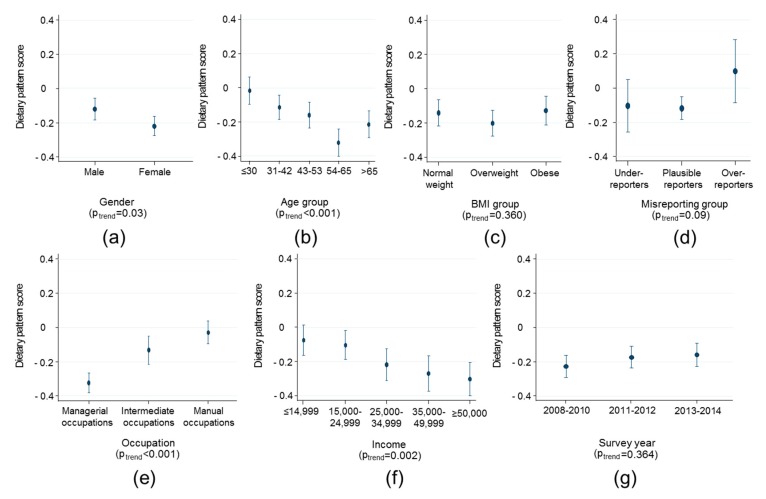
Trends in the obesogenic dietary pattern score among adults (>18 years) by (**a**) sex; (**b**) age; (**c**) weight status; (**d**) energy reporting status; (**e**) occupation; (**f**) household income; and (**g**) survey year. Figures present marginal means and tests for trend with survey-weighted linear regression adjusted for age, gender and daily energy intake.

Trends in the obesogenic dietary pattern by various factors are displayed in Figure 2 and Figure 3 for children and adults, respectively. Among children, a more obesogenic dietary pattern score was associated with being female, living in a household with manual occupations and a lower income. The difference in dietary pattern score was largest for extremes of household income where families with >£50,000 per annum had a pattern score 0.29 (0.18, 0.39) units lower than households with <£14999 per annum in income. There was weak evidence that dietary pattern scores were more obesogenic in 2013–2014 compared with 2008–2010 (by 0.12 (95% CI 0.06, 0.18) units, *p* = 0.04), but there was limited statistical evidence for a trend across all time-points (*p* = 0.08, Figure 2, panel g). There was no evidence of association of dietary pattern score with misreporting category, age or weight status. Among adults, a more obesogenic dietary pattern score was associated with being male, younger, living in a household with manual occupations and a lower income (Figure 3). The difference in dietary pattern score was largest for extremes of age (<30 years vs. >54 years 0.37 95% CI 0.23, 0.50) and household occupation where families with managerial occupations had a pattern score 0.31 (0.22, 0.41) units lower than households with manual occupations. There was no evidence of association of dietary pattern score with misreporting category, BMI or survey year.

**Figure 4:**
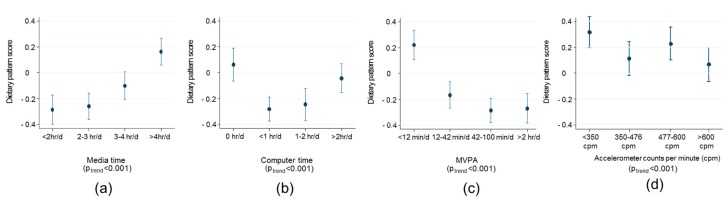
Trends in the obesogenic dietary pattern score among adults (>18 years *n* = 3549 panel a and b, *n* = 3790 panel (**c**) and children (aged 11–16 years, *n* = 861 panel d) by (**a**) media time; (**b**) computer time; (**c**) time spent in moderate to vigorous physical activity (MVPA); (**d**) objectively measured physical activity in counts per minute. Figures present marginal means and tests for trend with survey-weighted linear regression adjusted for age, gender and daily energy intake. Media time, computer time and MVPA have been assessed with the RPAQ questionnaire adults aged >18 years. Quartiles of these variables are presented in the current figure.

Strong trends were observed among adults in their obesogenic dietary pattern score by media time and time spent in MVPA (Figure 4). Adults spending less than 12 min/day in MVPA vs. more than 2 h/day had a more obesogenic dietary patterns score by 0.45 units (95% CI 0.30, 0.60). In addition, adults spending more than 4 h/day vs. less than 2 h/day watching media had a 0.41 unit (95% CI 0.27, 0.55) more obesogenic diet score. A similar inverse trend of a less obesogenic dietary pattern with increasing amounts of physical activity was observed in the small subsample of children with objectively measured physical activity from accelerometers (Figure 4, panel d). 

Among both adults and children, there was evidence that Northern Ireland has a more obesogenic diet than England (0.17 95% CI 0.10, 0.25 and 0.28 95% CI 0.16, 0.40 higher in Northern Irish children and adults compared with English respectively, Supplementary Figure S2). Associations between eating-related behaviours and obesogenic dietary pattern score are illustrated in Supplementary Tables S4 and S5 for children and adults, respectively. While eating out was not associated with pattern score, eating takeaways more than once a week compared with less than once a week was associated with a 0.19 (95% CI 0.12, 0.26) and 0.25 (95% CI 0.15, 0.36) more obesogenic diet in children and adults respectively. Being vegetarian (2% of the sample) was associated with a less obesogenic score by 0.63 (95% CI 0.45, 0.82) in children and 0.53 (95% CI 0.26, 0.81) units in adults. Data on dieting for weight loss was only available for 66% of the sample but indicated that adults on a diet to lose weight ate less obesogenic diets (−0.25 (95% CI 0.12, 0.38)). 

Our analysis derived an obesogenic dietary pattern in a nationally representative sample of UK adults and children for the first time and established its generalisability across a range of population subgroups. An energy-dense, low-fibre, high-fat pattern in the UK is characterised by low intakes of fruits, vegetables, high-fibre breakfast cereals, yoghurt and legumes and high intakes of chocolate, confectionery, low-fibre bread, biscuits, cakes and processed meat. We observed clear social gradients in the dietary pattern score, such that lower SES groups consumed the most obesogenic diets, highlighting a possible source of health inequalities. Among adults, an obesogenic diet was associated with more sedentary time and less time in MVPA, which could have important implications for energy balance.

Using a COC, we found that dietary patterns derived in different subgroups of the UK population were very similar to each other. Previous RRR derived dietary patterns identified in different populations sometimes showed fair agreement with the current results e.g., UK children and adolescents at age 7, 10 and 13 years in ALSPAC (COC–0.88, 0.88 and 0.90 respectively) [11], and with Australian adolescents participating in the National Children’s Nutrition and Physical Activity Survey (COC–0.92) [24]. However, more diverse pattern loadings were seen between our findings and European adolescents (COC–0.82) [24], Australian children (COC–0.83) [25] and German adults (COC–0.55) [7]. Comparability across studies is limited when different numbers and composition of food groups are used. Schulz et al. reported a simplified version of their dietary pattern, with only loadings for 10 food groups and Appannah et al. (2014), only had 23 food group loadings available for comparison [25]. Pattern comparability is also limited by the use of different intermediates, i.e., carbohydrate density rather than energy density [7]. Methodological differences are an important source of variation in the consistency of patterns, however real population variation in food intakes may also explain why diverse foods contribute to an obesogenic pattern in different countries. At least for the UK, it is clear that the pattern derived in the current analysis is appropriate for measuring the obesogenicity of diets among children, adults, and low and high socioeconomic groups alike.

Using the same dietary pattern score, we were able to observe important correlates of an obesogenic diet in the UK population, which was in line with groups with a higher prevalence of obesity. There were clear social gradients, which has also been observed in other studies deriving dietary patterns in the same way [10,22], and is in line with the more general observation that diet quality is socially patterned [26]. Interestingly there was no evidence of association between our dietary pattern score and weight status in either adults or children. The lack of evidence could be because of the cross-sectional design, which is limited for dissecting the temporal order of associations. It is likely that having obesity elicits changes to diet to make it less obesogenic in an attempt to control or lose weight, which is supported by our findings that adults on a weight loss diet had a lower pattern score. However, previous longitudinal studies of the association of this pattern with fat mass in children and adolescents supports that it is obesogenic [10,11]. Further prospective studies among adults and experiments are required to confirm if the pattern is also obesogenic in later life and that changing the current pattern score can prevent obesity.

A major strength of this study is that analyses were carried out in a large, nationally representative sample of ~10,000 adults and children in the UK. To our knowledge, this is the first study in the UK to have used a national survey to identify if food contributing to an obesogenic dietary pattern is the same in both children and adults. Using RRR to conduct the analysis allowed us to derive pattern loadings characteristic of intermediate dietary factors that have been associated with the development of obesity, namely DED, %fat and FD, thereby basing our findings on well-established diet-disease hypotheses [27]. One advantage of the current dietary pattern score, over individual foods/nutrients, is that it captures variation in multiple aspects of an obesogenic diet in a single score and offers a scale indicating the balance of foods likely to increase or decrease obesity. Incorporating our obesogenic diet score into computerised systems for monitoring food intake could offer a novel approach to measuring the success of individual or policy level obesity prevention.

The use of food records ensured a high level of detail on dietary intake was available to derive the dietary pattern. There is an ongoing debate regarding the validity and use of self-reported dietary data in research [28,29,30,31]. Although food records are not free of error in assessing habitual food consumption, they are completed prospectively in real-time and so avoid the reliance on recall commonly encountered with other memory-based methods in such as food frequency questionnaires and 24-h recalls [32]. Food records are still prone to a certain degree of under-reporting and reactivity or under-eating compared with habitual intakes, particularly among overweight individuals [33,34]. However, when compared with doubly labelled water and direct observation, food records consistently outperform other self-reported methods and capture on average 80% of true energy intake [35,36,37,38]. Differential under-reporting by BMI may explain the lack of evidence of a cross-sectional association between the dietary pattern and weight status. Therefore, we quantified misreporting of energy intake by comparing to estimated energy requirements and observed no evidence of association with the dietary pattern score, suggesting that error in the food records was non-differential in relation to the pattern score. Despite these strengths, including the consideration that many potential confounders were accounted for in analyses, as with any observational study the possibility of residual confounding cannot be ruled out. Self-reported physical activity was used to investigate trends with an obesogenic diet among adults as this was the most complete data available in NDNS. Validation studies have shown that questionnaire-based PA measurement, while being reliable, have only modest validity [39]. The RPAQ tends to overestimate MVPA and underestimate sedentary time but the ranking of participants is similar [21], which means that estimates of effect size may not be accurate, but the direction of associations should be indicative of the true association. We further investigated the association of pattern score and PA in a sub-sample of children in NDNS who had objective accelerometer-measured PA available and similar inverse trends were observed. While the size of the association may have been over estimated by the error in the questionnaire measurement, the consistency in the direction of the association between objective and subjective measures supports the conclusion that less PA is associated with a more obesogenic diet.

Our analysis used data spanning 6 years and 4 countries, allowing the exploration of differences in the ‘obesogenicity’ of diets across the UK and over time based on contemporary data on the dietary intake of the UK population [13]. Monitoring the pattern score over time could be a tool to measure the effectiveness of policy, environmental or individual changes to prevent obesity. The specific foods highlighted as most important to the pattern score could also inform strategies to reduce obesity prevalence in the UK by tackling the provision of foods that make diets more or less obesogenic. Specific sub-groups of people consuming a more obesogenic diet include low income and occupational classes, less active and more sedentary, young, male adults, and children in general (who had systematically higher dietary pattern score than adults). These sub-groups may benefit from targeted obesity prevention policies. The present findings have important implications for the development of personalised interventions to prevent obesity, by promoting dietary patterns characterised by foods low in energy density and fat, and high in dietary fibre. Having many different foods contributing to the obesogenic dietary pattern score provides a wide range of options for change that individuals can choose to make their diet less obesogenic. The current dietary pattern score might offer an important opportunity for the development of personalised approaches for the prevention of excess weight gain by enabling individuals to find an acceptable and bespoke menu of small changes to their food intake that may be more sustainable in the long-term. 

## 5. Conclusions

In the UK, an energy-dense, low-fibre, high-fat dietary pattern is similar to the pattern previously identified in prospective studies as obesogenic. An obesogenic dietary pattern is generalizable across UK population sub-groups and is consistently characterised by low intakes of fruits, vegetables, high-fibre breakfast cereals, yoghurt and legumes and high intakes of chocolate, confectionery, low-fibre bread, biscuits, cakes and processed meat. Younger, more sedentary and less active adults and children from low SES groups are consuming more obesogenic diets, putting them at high risk of excessive weight gain. Interventions based on modifying and monitoring this obesogenic pattern of food intake should be developed and tested as they could offer an innovative approach for the precision prevention of obesity. 

## Figures and Tables

**Table 1:** Description of the National Diet and Nutrition Survey children and adult sample in 2008–2014.

		Children (*N* = 4636)		Adults (*N* = 4738)	
		% N ^a^	95% CI	% N ^a^	95% CI
Gender	Male	51	(49, 53)	49	(47, 50)
	Female	49	(47, 51)	51	(50, 53)
Age group	1–5 years	27	(25, 28)		
	6–10 years	27	(25, 28)		
	11–18 years	47	(45, 49)		
	19–64 years			79	(77, 80)
	>65 years			21	(20, 23)
Occupation ^b^	Managerial & professional occupations	43	(40, 45)	44	(42, 46)
	Intermediate occupations	20	(19, 22)	21	(19, 22)
	Routine & manual occupations	37	(35, 39)	34	(32, 36)
Household income ^c^ (£)	<23,000	57	(54, 59)	46	(44, 48)
	≥23,000	43	(41, 46)	54	(52, 56)
Survey year	2008–2010	38	(34, 42)	37	(33, 41)
	2011–2012	35	(32, 39)	36	(32, 40)
	2013–2014	27	(24, 31)	27	(24, 31)
BMI ^d^	Normal weight	76	(74, 77)	37	(35, 39)
	Overweight	17	(16, 19)	37	(35, 39)
	Obese	7	(6, 8)	27	(25, 29)
Media time ^e^	≤3 h/day			47	(44, 49)
	>3 h/day			53	(51, 56)
Computer time ^e^	≤1 h/day			40	(38, 43)
	>1 h/day			60	(58, 62)
MVPA ^f^	≤36 min/day			46	(43, 48)
	>36 min/day			54	(52, 57)
Misreporting of energy intake	Under-reporters	9	(8, 10)	19	(17, 20)
	Plausible reporters	79	(77, 80)	71	(70, 73)
	Over reporters	12	(11, 13)	10	(9, 11)

Abbreviations: BMI–Body Mass Index, CI–Confidence Interval, MVPA–Moderate to Vigorous Physical Activity. ^a^ Percentages are weighed based on non-selection and non-response survey weights provided by NDNS; ^b^ Occupation is based on the National Statistics Socio-economic Class (NS-SEC). There was 2% missing data in children and 2% missing data in adults; ^c^ There was 11% and 14% missing data for household income in children and adults respectively; ^d^ BMI categories are based on international (IOTF) BMI cut-offs [19]. For children, BMI z-score were created by standardising BMI for sex and age based on the 1990 British Growth Reference (UK90) [20]). For adults, BMI is measured in kg/m^2^ and standard WHO cut-offs used to define weight status. There was 9% and 7% missing data in children and adults respectively; ^e^ There was 27% missing data in adults; ^f^ There was 20% missing data adults.

## Supplementary Materials

The following are available online at http://www.mdpi.com/2072-6643/10/4/388/s1, Figure S1: Dietary pattern loadings for each food group in different age groups; Figure S2: Mean dietary pattern score by each country in the UK in (a) adults (>18 years) and (b) children (<18 years); Table S1: Comparison of dietary patterns across sub-groups of the population; Table S2: Descriptive dietary characteristics by quintile (Q) of dietary pattern score in children (≤18 years); Table S3: Descriptive dietary characteristics by quintile (Q) of dietary pattern score in adults (>18 years); Table S4: Trends in dietary pattern by eating-related behaviours in children (≤18 years); Table S5: Trends in dietary pattern by eating-related behaviours in adults (>18 years); Information S1: Food groups and their contents; Information S2: Calculating a coefficient of congruence; Information S3: Code used to run RRR in stata.
