# Supplementary material for: Social Gradients and Physical Activity Trends in an Obesogenic Dietary Pattern: Cross-Sectional Analysis of the UK National Diet and Nutrition Survey 2008–2014
Source: Nutrients. 2018 Mar 22;10(4):388. doi: 10.3390/nu10040388 (PMC5946173; doi:10.3390/nu10040388)

Supplementary information 1: Food groups and their contents

1. High fat milk and cream
Whole milk, Channel Island milk, UHT whole milk

Elmlea (single, whipping and double), evaporated milk, single cream, whipping cream, double cream, clotted cream, UHT cream (half, single and whipping).

Flavoured milk (Horlicks made up with whole milk, milk shake powder made up with whole milk, Ovaltine powder made up with whole milk, drinking chocolate powder made up with whole milk, purchased milk shake, Horlicks powder made up with semi-skimmed milk, milkshake powder made up with semi-skimmed milk, Cocoa powder made up with semi-skimmed milk, Ovaltine made up with semi-skimmed milk, drinking chocolate powder made up with semi-skimmed milk, Ovaltine powder made up with skimmed milk, drinking chocolate powder made up with skimmed milk)

2. Low fat milk

Semi-skimmed milk, Dream Topping made up with semi-skimmed milk, skimmed milk, dried skimmed milk, Plain soya milk, flavoured soya milk, calcium-enriched soya milk.

3. Yoghurts
Drinking yoghurt, Greek yoghurt, Fruit Corner yoghurt, Crumble Corner yoghurt, organic whole milk yoghurt, plain and fruit fromage frais, Crunch Corner yoghurt, Greek yoghurt and honey, fromage frais ready meal, Luxury Corner yoghurt, low calorie yoghurt, plain and flavoured low-fat yoghurt, very low fat fromage frais, soya yoghurt.

4. Cheese
Brie, cheddar, vegetarian cheddar, cheese spread, cottage cheese, edam, feta, soft cheese, gouda, parmesan, processed cheese, flavoured cheese spread, double Gloucester, emmental, gruyere, Leicester, smoked processed cheese, low-fat cheddar, quark, cheese spread light, mozzarella.

5. Butter and animal fat

Butter, spreadable butter, Dripping, lard

6. Margarine and Vegetable oils
Low-fat non-polyunsaturated margarine, low-fat polyunsaturated margarines, full-fat polyunsaturated margarines, full-fat non-polyunsaturated margarine, sesame oil, sunflower oil, vegetable oil, olive oil,

7. Eggs and egg dishes
Raw egg, raw egg white, raw egg yolk, boiled eggs, fried eggs, poached eggs, scrambled eggs, plain omelette, cheese omelette, cheese and egg quiche, Scotch eggs, Quiche Lorraine, mushroom quiche, egg fried rice,

8. Low fibre bread
White bread, white toast, French stick, white rolls, hamburger buns, pitta bread, muffins, bagels, toasted crumpets,

9. High fibre bread
Brown bread, brown toast, brown rolls, granary bread, soft grain bread, softgrain toast

Wholemeal bread, wholemeal toast, wholemeal rolls, rye bread, Hovis, Hovis toast, crispbread, wheatgerm bread

10. Other bread products
Croissants, malt bread, currant bread, breadcrumbs, fried bread, garlic bread, Savoury pancakes, Yorkshire pudding, stuffing, dumplings, flaky pastry, shortcrust pastry, papadums, Naan bread, chappatis, wheatgerm, white flour, wholemeal flour, Cream crackers, wholemeal crackers, ritz crackers, water biscuits, cheddars biscuits, oatcakes, rice cakes, breadsticks.

11. High fibre breakfast cereals
All Bran, Bran Flakes, Common Sense Oat Branflakes, Fruit'n'Fibre, Muesli, Oat and Wheat Bran, Raisin Splitz, Shredded Wheat, Sultana Bran, Weetabix, Bran Buds, Weetaflakes, Weetos, Shreddies, Start, Honey Smacks, Nutrigrain, Ready Brek, Grapenuts, raw oatmeal, Porridge, Nestle Clusters, minibix chocolate, minibix banana, minibix fruit and nut

12. Other breakfast cereals
Coco Pops, Cornflakes, Crunchy Nut Cornflakes, Frosties, Rice Krispies, Ricicles, Special K, Golden Grahams

13. Rice, pasta and other grains
Brown rice, savoury rice, white rice, risotto, plain pilau,

Macaroni, noodles, spaghetti, wholemeal spaghetti, boiled lasagne, Super Noodles,

Bulgur wheat, couscous

14. Cereal based mixed meals
Macaroni cheese, pasta salad, canned ravioli, canned spaghetti, spaghetti canned in bolognaise sauce, pot savouries made up, canned macaroni cheese, Dairylea dunkers (various), Dairylea Lunchables (various).

15. Pizza
Pizza, frozen pizza, French bread pizza,

16. Biscuits and cakes
Digestives, chocolate digestives, reduced-sugar digestives, flapjacks, homemade biscuits, jaffa cakes, sandwich biscuits, semi-sweet biscuits, short-sweet biscuits, shortbread, iced biscuits, fruit biscuits e.g. garibaldi, chocolate fingers, full-coated chocolate biscuits, ice cream wafers, fig rolls, gingerbread men, sponge fingers/boudoir biscuits, macaroons, filled wafer biscuits, Coco Pops cereal bar, Frosties cereal bar, rusks, chewy cereal bars, crunchy cereal bars, Special K cereal bar

Battenburg cake, made-up cake mix, crispie cakes, fancy iced cakes individual, fruit cake (plain, rich, iced and wholemeal), gateau, Madeira cake, sponge cake, fatless sponge cake, jam filled sponge cake, chocolate mini rolls, Chelsea buns, currant buns, custard tarts, Danish pastries, jam doughnuts, ring doughnuts, Eccles cakes, hot cross buns, jam tarts, mince pies, fruit scones, plain scone, scotch pancakes, toasted teacakes, iced buns, cherry cake, chocolate cake, chocolate cake with butter icing, coconut cake, gingerbread, lardy cake, rock cakes, swiss roll, Welsh cakes, custard doughnuts, iced

ring doughnuts, éclairs, mincemeat tarts, fresh teacakes, chocolate muffin, fruit muffin, chocolate teacake, choux buns, waffles, pop tarts, malt loaf, chocolate croissant

17. Puddings
Christmas pudding, fruit crumble, wholemeal fruit crumble, sponge pudding, cheesecake, crème caramel, custard, canned custard, instant dessert, jelly, milk pudding, chocolate mousse, fruit mousse, canned rice pudding, trifle, apple crumble, sponge flan with fruit, sponge pudding (with dried fruit or jam or treacle), spotted dick, blancmange, fruit fool, sweet pancakes, blackcurrant pie, fruit pie, lemon meringue pie, treacle tart, meringue, meringue with cream, apple pie, bakewell tart, bread pudding.

18. Ice-creams
arctic roll, choc ice, chocolate nut sundae, cornetto, frozen ice cream desserts, dairy ice-cream (vanilla and flavoured), frozen yoghurt, non-dairy ice-cream (vanilla, flavoured, mixes and reduced calorie), sorbet, Twix ice-cream, Mars ice-cream, Bounty ice-cream, ice-cream with cone, Strawberry split, Kit Kat ice-cream.

19. Chocolate and confectionery
Boiled sweets, chew sweets, foam sweets, fruit gums/jellies, fruit pastilles, fudge, liquorice allsorts, liquorice shapes, marshmallows, nougat, peppermints, sherbet sweets, toffees, Turkish delight, dolly mixtures, chewing gum with sugar, marzipan, glacé cherries, candied popcorn, Chocolate covered caramels, chocolate covered bar with fruit/nut wafer, chocolate fancy and filled, milk chocolate, plain chocolate, white chocolate, Crème eggs, Kit Kat, Mars Bar, Milky Way, Smartie-type sweets, Snickers, Twix, Bounty Bar, whole nut chocolate, fruit and nut chocolate,White sugar, , Ice Magic Sauce, ice-cream sauce/topping, butter icing, glace icing, fondant icing, brown sugar, Demerara sugar, golden syrup, maple syrup, mincemeat

20. Sugar-free confectionery and desserts
Sugar-free chewing gum, sugar-free fruit gums, sugar-free mints, sugar-free instant dessert powder, sugar-free jelly, reduced sugar jam.

21. Spreads
Chocolate spread, chocolate nut spreads, jam, honey, lemon curd, marmalade, peanut butter, sandwich spread

22. Meat and poultry
Stewed beef mince, rump steak, silverside, sirloin joint, stewing steak, topside, beef steak pudding, stewed steak and kidney, veal cutlets, beef grillsteaks, pork and beef meatballs, lamb cutlets, roast lamb (leg and shoulder), lamb loin chops, lamb mince, stewing lamb, lamb burger, stewed pork, pork belly slices, hand of pork, leg of pork, pork loin chops, stewed pork mince, roast pork, pork spare ribs, roast duck, roast pheasant casseroled chicken (breast, leg and thighs), roast chicken (light meat, leg, wing, whole roast chicken), stewed turkey mince, roast turkey (light meat and average)

23. Meat mixed dishes

Beef curry, beef stew, Bolognese, chilli con carne, lasagne, chop suey with beansprouts and beef, cottage pie, beef hot pot, lamb bolognaise, moussaka, shepherds pie, Irish stew, pork casserole, sweet and sour pork, pork and chicken chow mein, pork curry, meat spread, corned beef hash, meat paste, pancake roll, meat school dinner, meat or fish mixed school dinner, savoury rissoles, Beef pie, chicken pie, Cornish pastie, pork pie, sausage rolls, steak and kidney pie, chicken pie, toad-in-the-hole, Chicken casserole with vegetables, , chicken curry, chicken in white sauce, chicken risotto, tandoori chicken, chicken pancakes, turkey bolognaise,

24. Processed meat
Back bacon, middle bacon, streaky bacon, gammon joint, gammon rashers, ham, bacon collar joint, bacon gammon joint, chopped ham and pork, luncheon meat, corned beef, garlic sausage, pepperami, polony, salami, Billy Bear meat, Chicken liver, lamb liver, ox liver, faggots, liver pate, low-fat meat pate, Beef burgers, hamburger, beef sausages, pork sausages, low-fat pork sausages, pork and beef sausages, frankfurter, black pudding, chicken/turkey sausages, chicken roll, turkey ham.

25. Coated or breaded meat and fish
Fish school meal, Cod in batter, cod in crumbs, haddock in crumbs, plaice in batter, scampi, squid in batter, fish cakes, home-made fish cakes, fish fingers, Chicken breast in crumbs, chicken Kiev, chicken burgers (takeaway), turkey burger.

26. Meat substitutes (Quorn and soya products)

Soya mince, vegebangers, vegeburgers, Quorn, Quorn Sausages/Burgers

27. Fish
Baked cod, poached cod, grilled cod, cod in parsley sauce, steamed haddock, prawns, fish paste, fish pie, kedgeree, tamarasalata, Smoked mackerel, canned mackerel, canned pilchards, steamed salmon, sardines, tuna, salmon fish cake, brown trout, tuna bake, whitebait

28. Fried/roast potatoes and chips
Roast potatoes, chips, potato waffles, French fries, microwave chips, oven chips, potato croquettes, potato waffles, school meal potatoes, fried potato cakes

29. Cooked potatoes
Boiled new potatoes, baked potatoes, boiled old potatoes, mashed potatoes, instant potato, gnocchi, duchesse potatoes

30. Vegetables (Raw or boiled)
Raw cabbage, raw red cabbage, raw white cabbage, raw celery, raw cucumber, endive, lettuce, iceberg lettuce, raw mushrooms, mustard and cress, raw onions, red peppers, yellow peppers, spring onions, watercress, green salad, raw beetroot, raw broccoli, raw garlic, raw red chillis, avocado, olives, pickled gherkins, pickled beetroot, Raw carrots (young and old), Raw tomatoes, cherry tomatoes. Canned bamboo shoots, beetroot, celeriac, celery, courgette, boiled fennel, boiled leeks, boiled onions, boiled parsnip, green peppers, red peppers, boiled plantain, boiled swede, baby sweetcorn, sweetcorn, corn-on-the-cob, ratatouille, boiled mushrooms, mangetout, frozen mixed vegetables, school meal vegetables, Boiled carrots (young, old and frozen), canned carrots, Tomato puree, grilled tomatoes, canned tomatoes. Globe artichokes, broccoli, purple broccoli, Brussels sprouts, red cabbage, savoy cabbage, cauliflower, curly kale, spinach, spring greens, asparagus, boiled cabbage, boiled amaranth leaves, turnip tops French beans, runner beans Mushy peas, peas, frozen peas, canned peas, petit pois, processed peas

31. Fried vegetables
Fried aubergines, fried courgette, fried mushrooms, fried onions, fried vegetable stirfry mix, stirfried beansprouts, fried tomatoes, roast parsnip

32. Legumes
Broad beans, butter beans, chick peas, green and brown lentils, red lentils, mung beans, red kidney beans, lentil roast, lentil and nut roast, hummus, Baked beans

33. Vegetable mixed dishes
Vegetable samosas, broccoli flan, cheese and mushroom flan, cheese, onion and potato flan, vegetable lasagne, vegetable pie, vegetable shepherds pie, vegetable pancake roll, cheese and potato pie, cauliflower cheese, cauliflower in white sauce, bubble and squeak, cheese and onion pastie, onion rings, pakoras, vegetable curry, vegetable pilau, vegetarian school meal, potato salad, coleslaw.

34. Fresh fruit
Clementines, grapefruit, lemons, oranges, satsumas, tangerines, Apples, peeled apples, Cox's apples, Golden Delicious, Granny Smith, dessert apples, pears, peeled pears, Bananas, Apricots, fresh coconuts, ready-to-eat apricots, blackberries, cherries, cranberries, currants, raw dates, fruit salad, grapes, kiwi fruit, lychees, mangoes, melon, Canteloupe melon, Galia melon, Honeydew melon, nectarines, passion fruit, peaches, pineapple, plums, yellow plums, pomegranate, prunes, ready-to-eat prunes, raspberries, frozen raspberries, redcurrants, strawberries, pure fruit ready meal, Fruit cocktail canned in juice, grapefruit canned in juice, peaches canned in juice, pineapple canned in juice

35. Other fruit
Stewed apple, baked apples, dried apples, banana chips, dried apricots, dried dates, dried mixed fruit, dried peaches, dried pineapple, stewed plums, raisins, stewed rhubarb, sultanas, Canned mandarin oranges, canned fruit cocktail, canned peaches, canned pears, canned pineapple, canned raspberries, canned strawberries, canned apricots

36. Nuts and seeds
Almonds, toasted almonds, Brazil nuts, cashew nuts, chestnuts, hazelnuts, peanuts, pine nuts, pistachio nuts, sesame seeds, sunflower seeds, toasted sunflower seeds, walnuts, creamed coconuts, dessicated coconuts.

37. Crisps and savoury snacks
Potato crisps, crinkle-cut crisps, low-fat crisps, square crisps, thick-cut crisps, potato and corn sticks, potato rings, pretzels, puffed potato products, tortilla chips, twiglets, wheat crunchies, corn snacks, corn and starch snacks, maize and rice flour snacks, mixed cereal and potato flour snacks, pork scratchings, plain popcorn, , Bombay mix

38. Soups
Carrot and orange soup, chicken soup, condensed chicken soup, chicken noodle soup, made-up instant soup, canned lentil soup, canned minestrone, creamed mushroom soup, canned oxtail soup, Scotch broth, canned tomato soup, condensed canned tomato soup, vegetable soup, canned vegetable soup

39. Sauces (low ED < 10 kJ/g)

Vinegar (0.9), Gravy (1.4), 'Fat free' dressing (1.6), Cook-in sauce (1.8), soy sauce (1.8), Canned sweet and sour sauce (1.9), Tomato-based pasta sauce (2.0), Tomato and mushroom sauce (2.5), Worcestershire sauce (2.7), Home-made tomato-based sauce (2.9), Canned curry sauce (3.2), Oyster sauce (3.4), Black bean sauce (3.5), Barbecue sauce (3.9), Tomato sauce (4), Brown sauce (4.2), Mint sauce (4.3), Bread sauce (4.6), Tomato ketchup (4.8), Smooth mustard (5.8), Wholegrain mustard (5.8), Sweet pickle (6.0), White pasta sauce. (6), White sauce (5.4-6.4), Cranberry sauce (6.5), Sweet and sour sauce (6.7), Salad cream Low cal (8.0), Cheese sauce (home-made and packet mix) (8/4), Apple sauce (8.1)

40. Sauces (high ED > 10 kJ/g)

Mayonnaise Low cal (11.9), Mango chutney (12.0), Salad cream (14.4), Blue cheese dressing (18.8), Mustard powder (18.8), French dressing (19.0), Pesto (21.4), Mayonnaise (28.4)

41. Condiments
Bovril, marmite, Oxo cubes, table salt, vegetable stock cubes, chicken stock cubes, Basil, bay leaf, chives, coriander, garlic powder, ground ginger, nutmeg, oregano, parsley, pepper, poppy seeds, rosemary, thyme, cinnamon, oregano

42. Sugar-sweetened beverages
Lime juice cordial, orange drink, undiluted ribena, fruit drink – high kcals, undiluted barley squash, fruit drink 300 kcals, fruit drink 48 kcals, Cola, carbonated fruit juice drinks, dry ginger ale, lemonade, lucozade, ready-to-drink fruit juice drink, made-up high juice drink, Ribena made up in carton, traditional lemonade, Sunny Delight, Lucozade sport, fruit drink 30kcals

43. Low energy beverages
Ribena lite undiluted, ribena sugar free, low calorie squash undiluted, Ribena toothkind, undiluted low-sugar high-juice drink, Diet cola, diet lemonade, caffeine-free diet cola, Ribena Toothkind made up in carton, ready-to-drink low-calorie fruit juice drink

44. Fruit juice
Apple juice, concentrated apple juice, grape juice, concentrated grape juice, lemon juice, canned mango juice, orange juice, concentrated orange juice, passion fruit juice, pineapple juice, mixed fruit juice, tomato juice

45. Hot and Powdered drinks
Bournvita powder, cocoa powder, drinking chocolate powder, Horlicks low-fat instant powder, Horlicks low-fat instant powder made up with water, milk shake powder, Ovaltine powder, milkshake syrup, powdered artificial sweetener.

Herbal tea, Tea, decaffeinated tea, Instant coffee granules, decaffeinated instant coffee granules, Coffee infusion, made-up instant coffee

46. Water
Mineral water, flavoured water, water

47. Alcoholic drinks

Wine, beer, spirits

48. Low alcohol and alcohol free beverages

Low alcohol wine, alcohol free beer

49. Commercial toddler foods

Baby biscuits, baby meals, infant yoghurts

50. Commercial toddler drinks

Infant fruit juices

51. Infant formulas

Infant type milks

Supplementary information 2: Calculating a coefficient of congruence

The coefficient of congruence (φ) is an indicator of the similarity of pattern loadings and is calculated with the formula below:

$$\varphi(x,\ y) = \frac{\sum x_i y_i}{\sqrt{\sum {y_i}^2 \sum {x_i}^2}}$$

where x_i and y_i are pattern loadings for each predictor variable i (n=1…51) on patterns x and y that are being compared, e.g. x=Dietary pattern for the total sample and y=Dietary pattern in boys.

Supplementary information 3: Code used to run RRR in stata
Reduced Rank Regression of Dietary Energy Density (ded), Fibre Density (fd) and percentage energy from fat (percent_fat) with all food groups (*ave_grams_foodgroup1-ave_grams_foodgroup51)*

```
plssas, y(ded fd percent_fat) x(ave_grams_foodgroup1-ave_grams_foodgroup51) method(rrr) exe(C:\Program Files\SASHome\SASFoundation\9.4\sas.exe)
insheet using  "N:\Stata output\out.csv", comma clear
save “PersonLevelFile_Y1-6_work_RRR.dta", replace
```

Supplementary table 1: Comparison of dietary patterns across sub-groups of the population

		N	Explained variation (%)					Coefficient of congruence[c]	Correlation Coefficient		
			Responses (total)	DED (kJ/MJ)	FD (g/MJ)	% fat	Food intake (total)		DED (kJ/MJ)	FD (g/MJ)	% fat
**Total sample**	Dietary pattern 1[a]	9374	43.9	59.1	44.7	27.8	3.3	-	0.8**	-0.7	0.5
	Dietary pattern 2[a]	9374	13.8	63.1	70	39.6	3.0	-	0.2**-	0.5	0.3
	Dietary Pattern 3[a]	9374	9.5	76	71.4	53.9	2.0	-	0.4**-	0.1	-0.4
**Gender**	Men	4299	41.3	54.5	40.3	29.2	2.8	0.99	0.7**	-0.6	0.5
	Women	5075	46.3	62.1	48.2	28.6	3.6	1.00	0.8**	-0.7	0.5
**Age (years)**	1-5	1366	43.9	50.5	44	37.3	2.7	0.94	0.7**	-0.7	0.6
	6-10	1225	44.2	60.4	40.2	32	2.8	0.94	0.8**	-0.6	0.6
	11-18	2045	37.8	59.1	19.1	35.3	2.4	0.93	0.8**	-0.4	0.6
	19-64	3662	47.3	62	49.4	30.6	3.8	0.98	0.8**	-0.7	0.6
	>65	1076	50.8	65.3	54.4	32.9	3.9	0.96	0.8**	-0.7	0.6
**Occupation[b]**	Managerial & professional occupations	3731	45.	62.5	47.9	25.4	3.4	0.99	0.8**	-0.7	0.5
	Intermediate occupations	1899	41.6	55.8	46.2	22.8	3.3	0.99	0.7**	-0.7	0.5
	Routine & manual occupations	3547	44.1	58.2	40.6	33.6	3.1	0.99	0.8**	-0.6	0.6
**Household income (£)**	<23,000	4106	42.8	56.3	42.4	29.7	3.1	0.99	0.8**	-0.7	0.5
	≥23,000	4066	44.6	61.1	46.4	26.2	3.2	0.99	0.8**	-0.7	0.5
**Random samples**	Random Sample 1	4687	43.8	58.8	44.6	28.1	3.3	1.00	0.8**	-0.7	0.5
	Random Sample 2	4687	44.1	59.4	44.9	27.9	3.2	1.00	0.8**	-0.7	0.5

Abbreviations: DED – Dietary Energy Density, FD – Fibre Density

[a]Dietary pattern derived from RRR with 51 predictor variables, e.g. food groups, and 3 response variables, i.e. DED, FD and %fat. For sub-groups only pattern 1 variation is reported

[b]Occupation categories are based on the National Statistics Socio-economic Class (NS-SEC).

[c]Coefficient of congruence compares pattern loadings for each food group from the pattern derived in each sub group compared with the loadings derived in the total sample. 0.85 is considered a reasonable cut-off to indicate a poor level of congruence.

1 Supplementary Figure 1: Dietary pattern loadings for each food group in different age groups.

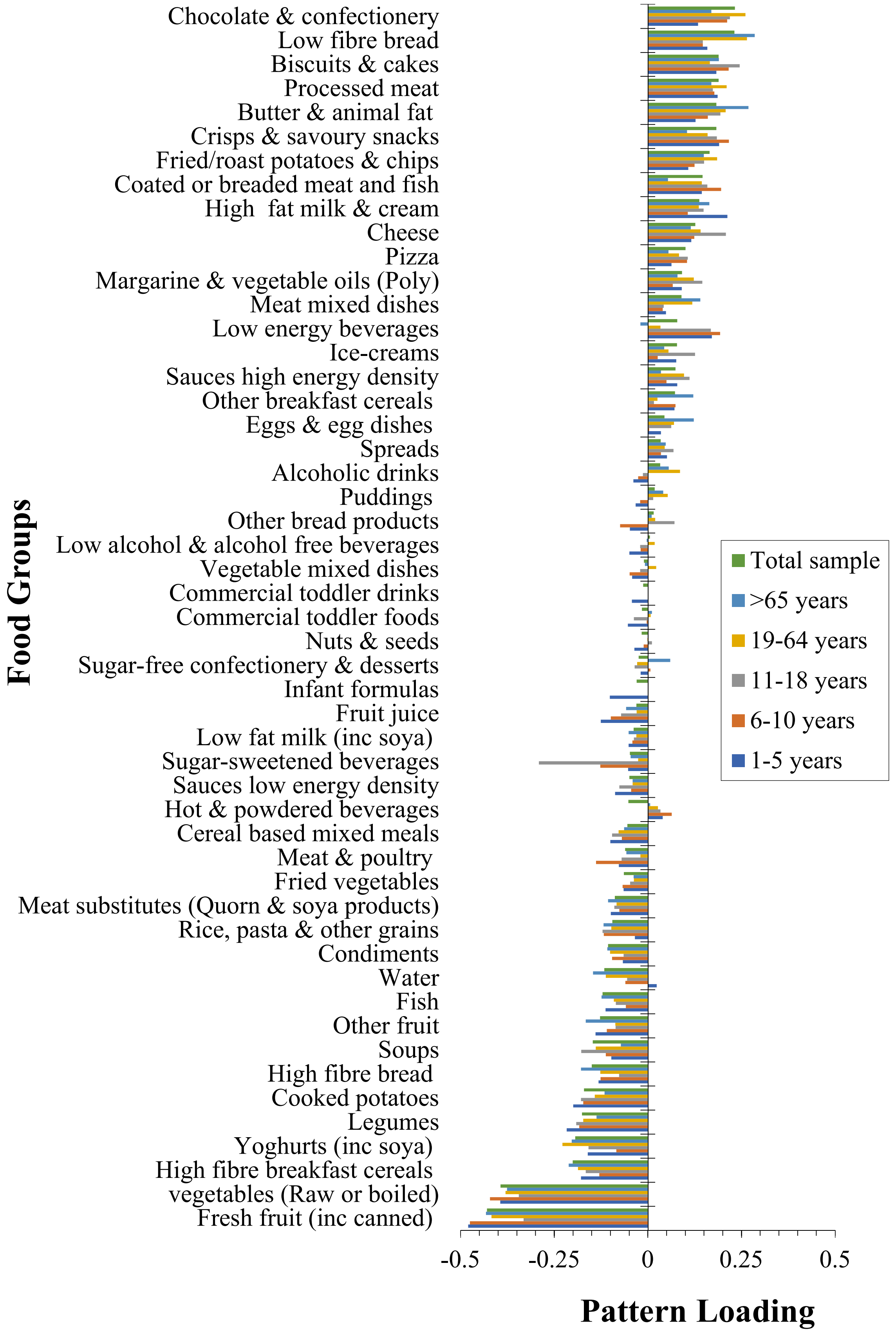

2

3

Supplementary table 2: Descriptive dietary characteristics by quintile (Q) of dietary pattern score in children (≤18 years).

	**Total sample**		**Q1**		**Q2**		**Q3**		**Q4**		**Q5**		**p-value[d]**
	**Mean**	**95% CI**	**Mean[c]**	**95% CI**	**Mean[c]**	**95% CI**	**Mean[c]**	**95% CI**	**Mean[c]**	**95% CI**	**Mean[c]**	**95% CI**	
**Dietary Pattern score**	0.1	(0.1, 0.2)	-1.4	(-1.5, -1.4)	-0.6	(-0.6, -0.5)	0.0	(0.0, 0.0)	0.5	(0.5, 0.5)	1.2	(1.2, 1.3)	<0.001
**DED (kJ/g)**	7.1	(7.1, 7.2)	5.3	(5.2, 5.4)	6.1	(6.0, 6.2)	6.8	(6.7, 6.9)	7.5	(7.5, 7.6)	8.6	(8.5, 8.8)	<0.001
**FD (g/MJ)**	1.7	(1.7, 1.7)	2.2	(2.1, 2.3)	1.9	(1.9, 1.9)	1.7	(1.7, 1.8)	1.6	(1.5, 1.6)	1.4	(1.4, 1.4)	<0.001
**% fat**	32.9	(32.7, 33.1)	28.0	(27.3, 28.7)	30.3	(29.9, 30.6)	32.4	(32.1, 32.7)	34.1	(33.8, 34.3)	36.4	(36.0, 36.7)	<0.001
**Energy intake (kcal/day)**	1577	(1559, 1595)	1554	(1501, 1607)	1514	(1482, 1547)	1491	(1461, 1521)	1555	(1528, 1582)	1775	(1737, 1813)	<0.001
	**%[ce]**	**95% CI**	**%[cf]**	**95% CI**	**%[cf]**	**95% CI**	**%[cf]**	**95% CI**	**%[cf]**	**95% CI**	**%[cf]**	**95% CI**	**p-value[d]**
**The weighted percentage of consumers from the whole sample or each quintile of pattern score for food groups that *decrease* the dietary pattern score[a]**													
Fresh fruit (including canned)	79	(77, 80)	88	(83, 92)	87	(83, 89)	84	(81, 87)	77	(74, 80)	62	(58, 66)	<0.001
Vegetables (Raw or boiled)	91	(90, 92)	96	(92, 98)	95	(93, 97)	93	(91, 95)	91	(89, 93)	83	(80, 86)	<0.001
High fibre breakfast cereals	51	(49, 53)	58	(52, 64)	60	(55, 64)	55	(52, 59)	45	(42, 49)	44	(40, 48)	<0.001
Yoghurts (including soya)	51	(49, 53)	61	(54, 67)	57	(53, 61)	56	(52, 60)	49	(45, 53)	40	(36, 44)	<0.001
Legumes	47	(45, 49)	65	(59, 72)	53	(48, 57)	53	(49, 57)	42	(38, 45)	34	(31, 38)	<0.001
**The weighted percentage of consumers from the whole sample or each quintile of pattern score for food groups that *increase* the dietary pattern score[b]**													
Chocolate & confectionery	84	(83, 86)	76	(70, 85)	80	(76, 84)	83	(81, 86)	85	(82, 88)	91	(88, 93)	<0.001
Low fibre bread	82	(81, 83)	72	(66, 77)	78	(74, 81)	(82	(79, 85)	82	(79, 85)	90	(87, 92)	<0.001
Biscuits & cakes	83	(81, 84)	70	(63, 75)	78	(74, 82)	81	(78, 84)	87	(84, 89)	89	(86, 91)	<0.001
Processed meat	78	(77, 80)	68	(61, 74)	76	(71, 79)	74	(70, 77)	81	(78, 84)	87	(84, 90)	<0.001
Butter & animal fat	34	(32, 35)	30	(24, 36)	30	(26, 34)	34	(30, 37)	34	(31, 38)	38	(34, 42)	0.04

Abbreviations: CI – Confidence Interval, DED – Dietary Energy Density, FD – Fibre Density [a]These are the top-5 food groups with the highest negative pattern loadings in the dietary pattern of the entire NDNS sample, i.e. food groups that decrease the obesogenic dietary pattern score. [b]These are the top-5 food groups with the highest positive pattern loadings in the dietary pattern of the entire NDNS sample, i.e. food groups that increase the obesogenic dietary pattern score. [c]Weighed means and percentages based on non-selection and non-response survey weights provided by NDNS. [d]Tests for trend with linear regression adjusted for age and sex. [e]Weighedproportions of children in the entire survey who are consumers of each food group. Percentages were calculated by dividing the number of children (≤18 years) consuming each food group by the total number of children in the survey, accounting for NDNS survey weights. [f]Weighedproportions of children within each quintile that are consumers of each food group. Percentages were calculated by dividing the number of children (≤18 years) consuming each food group by the total number of children within each quintile, accounting for NDNS survey weights.

Supplementary t**able 3:** Descriptive dietary characteristics by quintile (Q) of dietary pattern score in adults (>18 years).

	**Total sample**		**Q1**		**Q2**		**Q3**		**Q4**		**Q5**		**p-value[d]**
	**Mean[c]**	**95% CI**	**Mean[c]**	**95% CI**	**Mean[c]**	**95% CI**	**Mean[c]**	**95% CI**	**Mean[c]**	**95% CI**	**Mean[c]**	**95% CI**	
**Dietary Pattern score**	-0.2	(-0.2, -0.1)	-1.7	(-1.8, -1.7)	-0.6	(-0.6, -0.6)	-0.1	(-0.1, 0.0)	0.5	(0.5, 0.5)	1.4	(1.3, 1.4)	<0.001
**DED (kJ/MJ)**	6.7	(6.7, 6.8)	5.3	(5.2, 5.4)	6.1	(6.1, 6.2)	6.8	(6.7, 6.9)	7.4	(7.3, 7.5)	8.4	(8.3, 8.5)	<0.001
**FD (g/MJ)**	1.9	(1.9, 2.0)	2.5	(2.5, 2.6)	2.1	(2.0, 2.1)	1.9	(1.8, 1.9)	1.7	(1.6, 1.7)	1.4	(1.4, 1.5)	<0.001
**% fat**	33.9	(33.6, 34.1)	29.1	(28.6, 29.6)	32.6	(32.2, 33.1)	34.4	(33.9, 34.8)	36.0	(35.6, 36.4)	38.2	(37.7, 38.7)	<0.001
**Energy intake (kcal/day)**	1820	(1798, 1842)	1766	(1727, 1805)	1693	(1652, 1734)	1777	(1734, 1821)	1808	(1767, 1850)	2063	(2009, 2117)	<0.001
	**%[ce]**	**95% CI**	**%[cf]**	**95% CI**	**%[cf]**	**95% CI**	**%[cf]**	**95% CI**	**%[cf]**	**95% CI**	**%[cf]**	**95% CI**	**p-value[d]**
**The weighted percentage of consumers from the whole sample or each quintile of pattern score for food groups that decrease the dietary pattern score[a]**													
**Fresh fruit (including canned)**	77	(75, 78)	94	(92, 96)	86	(82, 88)	78	(74, 81)	66	(62, 70)	56	(51, 60)	<0.001
**Vegetables (Raw or boiled)**	96	(95, 97)	99	(99, 100)	99	(97, 99)	98	(96, 99)	94	(91, 96)	91	(88, 93)	<0.001
**High fibre breakfast cereals**	51	(49, 53)	67	(54, 71)	59	(54, 63)	49	(45, 54)	40	(36, 45)	34	(30, 38)	<0.001
**Yoghurts (including soya)**	39	(37, 40)	58	(54, 61)	45	(41, 49)	34	(30, 38)	31	(27, 36)	21	(18, 25)	<0.001

**Legumes**	41	(40, 43)	56	(52, 60)	46	(42, 50)	38	(33, 42)	35	(31, 39)	30	(26, 34)	<0.001
**The weighted percentage of consumers from the whole sample or each quintile of pattern score for food groups that decrease the dietary pattern score[b]**													
**Chocolate & confectionery**	77	(75, 78)	66	(62, 70)	73	(69, 77)	81	(77, 84)	81	(77, 84)	85	(82, 88)	<0.001
**Low fibre bread**	75	(73, 76)	58	(54, 62)	69	(65, 73)	77	(74, 81)	83	(79, 86)	90	(88, 92)	<0.001
**Biscuits & cakes**	73	(71, 74)	67	(62, 70)	71	(67, 74)	70	(67, 75)	76	(72, 79)	80	(77, 83)	<0.001
**Processed meat**	75	(74, 77)	63	(60, 67)	71	(67, 75)	79	(75, 82)	80	(77, 83)	86	(82, 89)	<0.001
**Butter & animal fat**	42	(40, 44)	37	(33, 40)	39	(35, 43)	44	(40, 48)	43	(39, 48)	50	(46, 55)	<0.001

Abbreviations: CI – Confidence Interval, DED – Dietary Energy Density, FD – Fibre Density [a]These are the top-5 food groups with the highest negative pattern loadings in the dietary pattern of the entire NDNS sample, i.e. food groups that decrease the obesogenic dietary pattern score. [b]These are the top-5 food groups with the highest positive pattern loadings in the dietary pattern of the entire NDNS sample, i.e. food groups that increase the obesogenic dietary pattern score. [c]Weighed means and percentages based on non-selection and non-response survey weights provided by NDNS.[d]Tests for trend with linear regression adjusted for age and sex. [e]Weighedproportions of adults in the entire survey who are consumers of each food group. Percentages were calculated by dividing the number of adults (>18 years) consuming each food group by the total number of adults in the survey, accounting for NDNS survey weights. [f]Weighedproportions of adults within each quintile that are consumers of each food group. Percentages were calculated by dividing the number of adults (>18 years) consuming each food group by the total number of adults within each quintile, accounting for NDNS survey weights.

Supplementary **table 4**.Trends in dietary pattern by eating-related behaviours in children (≤18 years).

		%N[a]	95% CI		Mean[a]	95% CI		p-value[b]
Eating out	Less than once a week	80	(79,	82)	0.14	(0.11,	0.18)	0.481
	Once a week or more	20	(18,	21)	0.17	(0.1,	0.23)	
Eating takeaway meals	Less than once a week	79	(78,	81)	0.11	(0.07,	0.14)	<0.001
	Once a week or more	21	(19,	22)	0.3	(0.24,	0.36)	
Vegetarian diet	Yes	2	(2,	3)	-0.47	(-0.66,	-0.29)	<0.001
	No	98	(97,	98)	0.16	(0.13,	0.19)	
Dieting[c]	Yes	7	(5,	9)	0.27	(0.07,	0.47)	0.792
	No	93	(91,	95)	0.24	(0.17,	0.31)	

[a]Weighed percentages and mean dietary pattern score based on non-selection and non-response survey weights provided by NDNS. [b]Test for trend with linear regression adjusted for age, gender and daily energy intake. [c]There was 72% missing data in this variable.

Supplementary **table 5**. Trends in dietary pattern by eating-related behaviours in adults (>18 years).

		%N[a]	95% CI		Mean[a]	95% CI		p-value[b]
Eating out	Less than once a week	73	(71,	74)	-0.17	(-0.22,	-0.12)	0.777
	Once a week or more	27	(26,	29)	-0.18	(-0.26,	-0.1)	
Eating takeaway meals	Less than once a week	79	(78,	81)	-0.22	(-0.27,	-0.18)	<0.001
	Once a week or more	21	(19,	22)	0.03	(-0.06,	0.12)	
Vegetarian diet	Yes	2	(2,	3)	-0.7	(-0.96,	-0.42)	<0.001
	No	98	(97,	98)	-0.16	(-0.2,	-0.12)	
Dieting[c]	Yes	17	(15,	18)	-0.37	(-0.49,	-0.25)	<0.001
	No	83	(82,	85)	-0.12	(-0.17,	-0.06)	

[a]Weighed percentages and mean dietary pattern score based on non-selection and non-response survey weights provided by NDNS. [b]Test for trend with linear regression adjusted for age, gender and daily energy intake. [c]There was 34% missing data in this variable.

Supplementary figure 2: Mean dietary pattern score by each country in the UK in (a) adults (>18 years) and (b) children (≤18 years).

Figures present margin means and tests for contrasts with survey-weighted linear regression adjusted for age, gender and daily energy intake. Adults n=4378; Children n=4636.

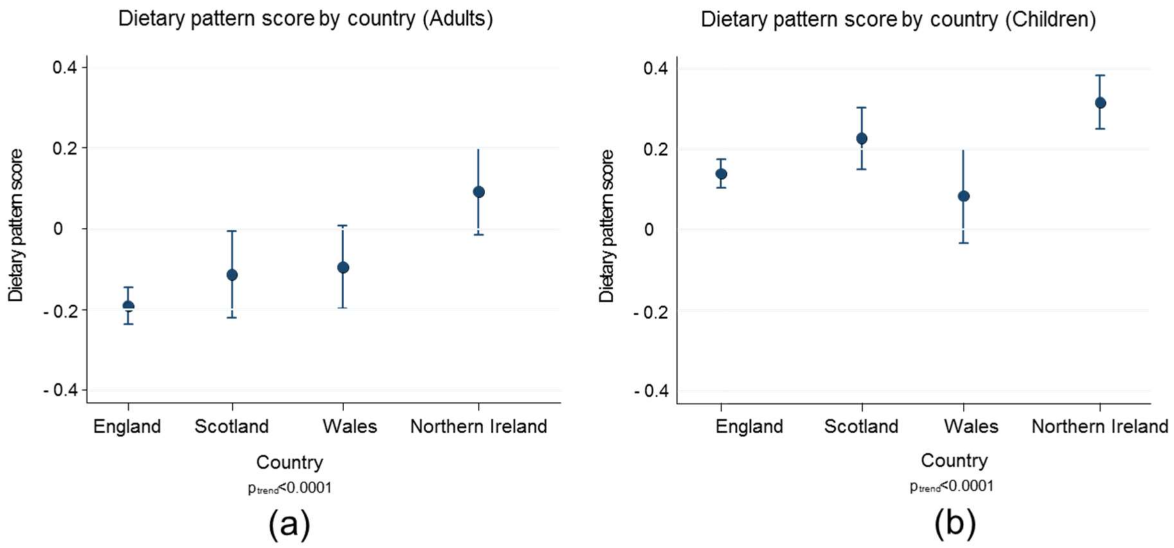

Supplement: Supplementary file 1 [file nutrients-10-00388-s001.pdf]
